# Supplementary material for: HIVprotI: an integrated web based platform for prediction and design of HIV proteins inhibitors
Source: J Cheminform. 2018 Mar 9;10:12. doi: 10.1186/s13321-018-0266-y (PMC5845081; doi:10.1186/s13321-018-0266-y)
Supplement: Supplementary file 2 — Additional file 2. Source code of HIVProtI web server. [file 13321_2018_266_MOESM2_ESM.zip › HIVprotI_Source-code/hivprotI/weka-3-6-6/doc/weka/classifiers/functions/pace/DiscreteFunction.html]

DiscreteFunction


---


|  |  |  |
| --- | --- | --- |
| **Overview** \| **Package** \| **Class** \| **Tree** \| **Deprecated** \| **Index** \| **Help** \| **Weka's home** | |  |
| **PREV CLASS**   **NEXT CLASS** | **FRAMES**    **NO FRAMES**     **All Classes** |  |
| SUMMARY: NESTED \| FIELD \| CONSTR \| METHOD | DETAIL: FIELD \| CONSTR \| METHOD |  |


---


## weka.classifiers.functions.pace Class DiscreteFunction

```
java.lang.Object
  weka.classifiers.functions.pace.DiscreteFunction
```

**All Implemented Interfaces:**: RevisionHandler

---

``` public class DiscreteFunction extends java.lang.Object implements RevisionHandler ```

Class for handling discrete functions.

A discrete function here is one that takes non-zero values over a finite
set of points.

**Version:**
:   $Revision: 1.4 $

**Author:**
:   Yong Wang (yongwang@cs.waikato.ac.nz)

---

**Constructor Summary**

`DiscreteFunction()`
  
          Constructs an empty discrete function

`DiscreteFunction(DoubleVector p)`
  
          Constructs a discrete function with the point values provides and the
function values are all 1/n.

`DiscreteFunction(DoubleVector p,
DoubleVector v)`
  
          Constructs a discrete function with both the point values and
function values provided.


| **Method Summary** | |
| --- | --- |
| `java.lang.Object` | `clone()`             Clones the discrete function |
| `double` | `getFunctionValue(int i)`             Gets a particular function value |
| `double` | `getPointValue(int i)`             Gets a particular point value |
| `java.lang.String` | `getRevision()`             Returns the revision string. |
| `boolean` | `isEmpty()`             Returns true if it is empty. |
| `static void` | `main(java.lang.String[] args)` |
| `DiscreteFunction` | `normalize()`             Normalizes the function values with L1-norm. |
| `DiscreteFunction` | `plus(DiscreteFunction d)`             Returns the combined of two discrete functions |
| `DiscreteFunction` | `plusEquals(DiscreteFunction d)`             Returns the combined of two discrete functions. |
| `void` | `setFunctionValue(int i, double v)`             Sets a particular function value |
| `void` | `setPointValue(int i, double p)`             Sets a particular point value |
| `int` | `size()`             Returns the size of the point set. |
| `void` | `sort()`             Sorts the point values of the discrete function. |
| `DiscreteFunction` | `timesEquals(double x)`             All function values are multiplied by a double |
| `java.lang.String` | `toString()`             Converts the discrete function to string. |
| `DiscreteFunction` | `unique()`             Makes each individual point value unique |

**Methods inherited from class java.lang.Object**

`equals, getClass, hashCode, notify, notifyAll, wait, wait, wait`

**Constructor Detail**

### DiscreteFunction

```
public DiscreteFunction()
```

:   Constructs an empty discrete function

---


### DiscreteFunction

```
public DiscreteFunction(DoubleVector p)
```

:   Constructs a discrete function with the point values provides and the
    function values are all 1/n.

    **Parameters:**: `p` - the point values

---


### DiscreteFunction

```
public DiscreteFunction(DoubleVector p,
                        DoubleVector v)
```

:   Constructs a discrete function with both the point values and
    function values provided.

    **Parameters:**: `p` - the point values: `v` - the function values


**Method Detail**

### normalize

```
public DiscreteFunction normalize()
```

:   Normalizes the function values with L1-norm.

---


### sort

```
public void sort()
```

:   Sorts the point values of the discrete function.

---


### clone

```
public java.lang.Object clone()
```

:   Clones the discrete function

    :   **Overrides:**: `clone` in class `java.lang.Object`

---


### unique

```
public DiscreteFunction unique()
```

:   Makes each individual point value unique

---


### size

```
public int size()
```

:   Returns the size of the point set.

---


### getPointValue

```
public double getPointValue(int i)
```

:   Gets a particular point value

    :   **Parameters:**: `i` - the index

---


### getFunctionValue

```
public double getFunctionValue(int i)
```

:   Gets a particular function value

    :   **Parameters:**: `i` - the index

---


### setPointValue

```
public void setPointValue(int i,
                          double p)
```

:   Sets a particular point value

    :   **Parameters:**: `i` - the index

---


### setFunctionValue

```
public void setFunctionValue(int i,
                             double v)
```

:   Sets a particular function value

    :   **Parameters:**: `i` - the index

---


### isEmpty

```
public boolean isEmpty()
```

:   Returns true if it is empty.

---


### plus

```
public DiscreteFunction plus(DiscreteFunction d)
```

:   Returns the combined of two discrete functions

    :   **Parameters:**: `d` - the second discrete function **Returns:**: the combined discrte function

---


### plusEquals

```
public DiscreteFunction plusEquals(DiscreteFunction d)
```

:   Returns the combined of two discrete functions. The first function is
    replaced with the new one.

    :   **Parameters:**: `d` - the second discrete function **Returns:**: the combined discrte function

---


### timesEquals

```
public DiscreteFunction timesEquals(double x)
```

:   All function values are multiplied by a double

    :   **Parameters:**: `x` - the multiplier

---


### toString

```
public java.lang.String toString()
```

:   Converts the discrete function to string.

    :   **Overrides:**: `toString` in class `java.lang.Object`

---


### getRevision

```
public java.lang.String getRevision()
```

:   Returns the revision string.

    :   **Specified by:**: `getRevision` in interface `RevisionHandler`
    :   **Returns:**: the revision

---


### main

```
public static void main(java.lang.String[] args)
```


---


|  |  |  |
| --- | --- | --- |
| **Overview** \| **Package** \| **Class** \| **Tree** \| **Deprecated** \| **Index** \| **Help** \| **Weka's home** | |  |
| **PREV CLASS**   **NEXT CLASS** | **FRAMES**    **NO FRAMES**     **All Classes** |  |
| SUMMARY: NESTED \| FIELD \| CONSTR \| METHOD | DETAIL: FIELD \| CONSTR \| METHOD |  |


---
